# A Mesoscale Study on the Dilation of Actively Confined Concrete under Axial Compression

**DOI:** 10.3390/ma15186490

**Published:** 2022-09-19

**Authors:** Peng Chen, Xiaomeng Cui, Huijun Zheng, Shengpu Si

**Affiliations:** 1 Shaanxi Key Laboratory of Safety and Durability of Concrete Structures, Xijing University, Xi’an 710123, China; 2 Department of Engineering Mechanics, Hebei Key Laboratory of Mechanics of Intelligent Materials and Structures, Shijiazhuang Tiedao University, Shijiazhuang 050043, China; 3 Department of Construction Engineering, Hebei Petroleum University of Technology, Chengde 067000, China

**Keywords:** dilation property of concrete in active confinement, secant strain ratio, lateral–axial strain relationship, mesoscale model of concrete

## Abstract

The confinement of concrete enhances its strength and ductility by restraining lateral dilation. The accuracy of a confinement model depends on how well it captures the dilation of concrete. In the current paper, a mesoscale model is established to study the dilation properties of concrete in active confinement, where the heterogeneity of concrete is considered. The stress–strain and lateral–axial strain curves of concrete in active confinement were used to demonstrate the validity of the mesoscale model. Subsequently, the distribution of lateral strain and the influences of the strength grade and confinement ratio on the dilation of concrete were investigated in a simulation. The results show that the distribution of the lateral strain along the radial or longitudinal directions is not uniform on the specimen when compressive failure occurs. The confinement ratio has a more significant influence on the concrete’s transverse dilation than the strength grade. Finally, an expression of the lateral–axial strain relationship of concrete in active confinement is proposed. The proposed formula can reflect the simulation results of the mesoscale model and is in good agreement with the prediction of existing formulas.

## 1. Introduction

The mechanical properties of confined concrete have been extensively studied in recent years, illustrating that confinement can enhance concrete’s strength and ductility by inhibiting transverse dilation [1,2,3,4]. Many practical design situations, such as the anchorage of pre-stressing reinforcement, bridge piles, containment vessels, and columns of high-rise buildings, require the prediction of concrete behavior under multiaxial stress states [5]. According to the loading path of confining pressure, the confinement can be divided into passive and active confinement [4,6]. In passively confined concrete, the confining pressure increases with the development of transverse dilation as well as restraining the transverse dilation of concrete. Thus, the key to predicting concrete properties in passive confinement lies in constructing a relationship between transverse dilation and confining pressure. Nevertheless, the development trend of confining pressure is greatly influenced by the type of confining material, which makes the study more complicated. In comparison to passively confined concrete, the force in actively confined concrete is relatively simple, in which the confining pressure remains constant. Therefore, it is more convenient to study the relationship between transverse dilation and confining pressure. Moreover, the relevant study can be applied to study the dilation of passively confined concrete by changing the confining pressure. The researchers have also adopted the same method to study the mechanical behavior of passively confined concrete [1,2,4,6,7,8].

The properties of concrete in active confinement have been experimentally studied recently [5,9,10,11]. However, the study focused more on the mechanical properties, while the study of dilation properties in the lateral direction is limited. Candappa et al. [12] tested the stress–lateral strain curves of concrete in active confinement. The analysis showed that the stress ratio versus the secant strain ratio was independent of the concrete strength and confinement ratio, and then a simplified formula was proposed. Binici [3] proposed a dilation model for concrete in active confinement, in which, the secant strain ratio remains constant in the elastic stage and tends to the limit value with the increase in the axial strain. Subsequently, another two lateral–axial strain models [13,14] were proposed by analyzing the experimental results of confined and unconfined concrete, in which the influence of confining pressure was explicitly introduced. The comparative analysis showed that the existing dilation models presented differences in form and the prediction results, which is caused by the selection of different test data. In addition, the measurement of strain presents a considerable discreteness, which is easily affected by the equipment, personnel, and testing method. Therefore, it is far from adequate to investigate the dilation property of confined concrete from the limited test data. The development of high-performance computers has facilitated mesoscopic simulations, which can consider the heterogeneous properties of concrete and the interaction among phases. The mesoscopic simulation can overcome the shortcomings of tests, such as the systematic error and the sensitive problem of strain measurement, which provide a new way to study the dilation behavior of concrete. To the best of the authors’ knowledge, no relevant studies have been conducted from this perspective. As an important supplement to the experimental study, the mesoscopic simulation conducted in this paper expands our understanding of concrete dilation.

This paper aims to develop a new method for studying the dilation properties of concrete in active confinement. To accomplish this objective, the concrete is regarded as a combination of the mortar phase, aggregate phase, and interfacial transition zone (ITZ), and then, a numerical concrete model is developed according to the theory of random distribution. The validity of the mesoscale model is first verified, and then a parameter analysis is conducted to study the influences of strength grade and confinement ratio on the dilation properties. Based on the simulation results, the lateral–axial strain curve of the concrete column in active confinement is proposed, and then it is compared to the existing models presented in the literature.

## 2. Mesoscale Model of Concrete

### 2.1. Random Aggregate Structure

Considering the heterogeneity of concrete at the mesoscale, existing studies [15,16,17,18,19,20] have shown that mesoscopic simulation is an effective method for investigating concrete’s mechanical performance. Thus, the dilation properties of concrete in active confinement were also studied using the same method.

The “take and place” method was used to generate aggregate particles, which was similar to the previous studies [21,22]. The volume content of coarse aggregate within each grading segment was determined according to the particle distribution curve proposed by Fuller [23]. Then, the coarse aggregate was randomly placed into the specimen’s size range under the premise that it did not coincide with the previous aggregate. In reality, the content of coarse aggregate is about 40–45% [24]. However, according to the studies conducted by Donza et al. [25] and Meddah et al. [26], relatively small coarse aggregate particles (less than 5 mm) were assumed to be contained in mortar to reduce the computational cost. The same treatment and proportional content are also used in the literature [27,28], and good prediction results have been achieved. Therefore, the volume of coarse aggregate was set as 30% of the concrete volume in the current study. The maximum aggregate size was 25 mm. The coarse aggregate with a particle size less than 5 mm was considered as the mortar, together with hardened cement, fine aggregate, voids, and micro-cracks. To improve the computation efficiency, 2 mm mesh was utilized to describe the ITZ around the particles.

**Figure 1:**
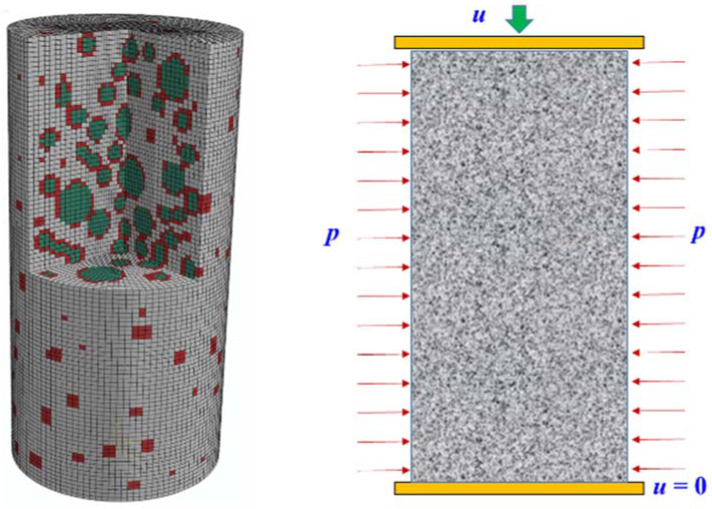
Random aggregate structure and loading method of concrete specimen.

Figure 1 reflects the random aggregate structure of concrete established through the method described above, and the height-diameter ratio is two. In this figure, the gray zone represents the mortar phase, the red zone denotes the ITZ phase, and the green area represents the aggregate phase. During the simulation, the interaction between the concrete specimen and loading plate was set as surface-to-surface contact, and the movement of the lower loading plate was fixed. To investigate the dilation properties of concrete in active confinement, the radial confining pressure *p* and vertical displacement *u* were applied to the specimen and upper loading plate, respectively.

### 2.2. Material Model

**Figure 2:**
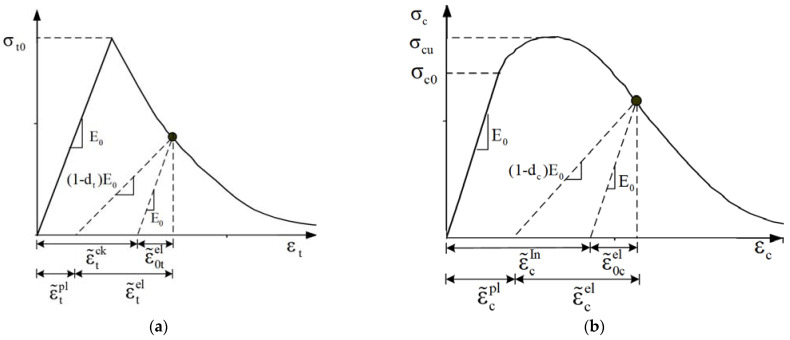
Concrete damage plasticity model. (**a**) Tensile stress–strain curve; (**b**) compressive stress–strain curve.

The damage plasticity model developed by Lee and Fenves [29] is extensively used in concrete definition, which has been incorporated into the software of ABAQUS. In this model, the failure of concrete can be divided into tension cracking and compression crushing, and two isotropic damage coefficients are introduced to describe the stiffness degradation of concrete. Figure 2 presents the damage plasticity model of concrete in uniaxial tension and uniaxial compression, respectively. In conditions of uniaxial tension, the stress–strain curve grows linear before reaching the tensile strength. Then, strain-softening occurs, and the tensile stiffness degrades. In conditions of uniaxial compression, the stress–strain curve is linear before reaching the proportional limit, then the plastic strain starts to develop, and the compressive stiffness begins to degrade. The relationship between stress and strain under uniaxial loading is defined by Equations (1) and (2) [29], in which, $d_{t}$, $\varepsilon_{t}$ and ${\tilde{\varepsilon }}_{t}^{\mathrm{pl}}$ represent the damage coefficient, the total strain, and plastic strain under axial tension, respectively. Similarly, the subscript c in the symbol represents the variables under axial compression.

(1) $$\sigma_{t}=\left(1-d_{t}\right)E_{0}\left(\varepsilon_{t}-{\tilde{\varepsilon }}_{t}^{\mathrm{pl}}\right)$$

(2) $$\sigma_{c}=\left(1-d_{c}\right)E_{0}\left(\varepsilon_{c}-{\tilde{\varepsilon }}_{c}^{\mathrm{pl}}\right)$$

Grote [30] highlighted that concrete and mortar have similar mechanical properties, thus the plastic damage model is also suitable for the mortar phase. Due to the high porosity of the ITZ phase, it can be assumed to be the mortar phase with reduced strength. Similar to the previous studies [31,32,33], the coefficient of 0.8 was used to describe the strength relationship between the ITZ and mortar phases. The coarse aggregate was usually regarded as the elastic body in the mesoscopic simulation accounting for its high strength and modulus. In general, the key of the material model lies in the definition of the mortar phase.

Experimental results show that the elastic modulus, peak strain, and decreasing index of the mortar phase differ from those of concrete with the same strength [34]. A parameter analysis was conducted in the authors’ previous work [35], and three correction constants were proposed to account for the differences. Finally, the constitutive relationship of the mortar phase is presented by Equations (3) and (4). It should be noted that the tensile failure of concrete was defined in terms of fracture energy, and the stress–cracking relation instead of the stress–strain relation was adopted to avoid mesh-size dependency [19,36].

(3) $$y=\left\{\begin{array}{c} 2x-x^{2}\\ \frac{xr}{r-1+x^{r}} \end{array}\right.\begin{array}{cc} & x\leq 1\\ & x>1 \end{array}$$

where $y=\sigma_{m}/f_{m}$, $x=\varepsilon_{m}/\varepsilon_{0}$, $\varepsilon_{0}=\left\{\begin{array}{c} \varepsilon_{m0}+x\left(\varepsilon_{\mathrm{mc}}-\varepsilon_{0}\right)\\ \varepsilon_{\mathrm{mc}} \end{array}\right.\begin{array}{cc} & x\leq 1\\ & x>1 \end{array}$, $\varepsilon_{m0}=1260+310\sqrt{f_{m}}$, $\varepsilon_{\mathrm{mc}}=\left(1+15p/f_{m}\right)\varepsilon_{m0}$; $r=E_{m}/\left(E_{m}-E_{p}\right)$, $E_{m}=4000\sqrt{f_{m}}$, $E_{p}=1.44f_{m}/\varepsilon_{m0}$.

(4) $$\sigma_{t}=\left\{\begin{array}{ccc} f_{t}\left(1-\frac{w}{w_{0}}\right) & & w\leq w_{0}\\ 0 & & w>w_{0} \end{array}\right.$$

where $w_{0}=2G_{F}/f_{t}$, $f_{t}=1.4{\left(f_{m}/10\right)}^{2/3}$, $G_{F}=G_{F0}{\left(f_{m}/10\right)}^{0.7}$ [37].

In a word, the elastic and plastic damage models were used for the aggregate and mortar phases in the random aggregate structure. Meanwhile, the ITZ phase can be regarded as a mortar phase with reduced strength. The main parameters of the constitutive model include the compressive strength of mortar *f*_m_, and the confining pressure *p*. Table 1 presents the mechanical properties of each phase.

### 2.3. Strength Relationship between Concrete and Mortar

**Figure 3:**
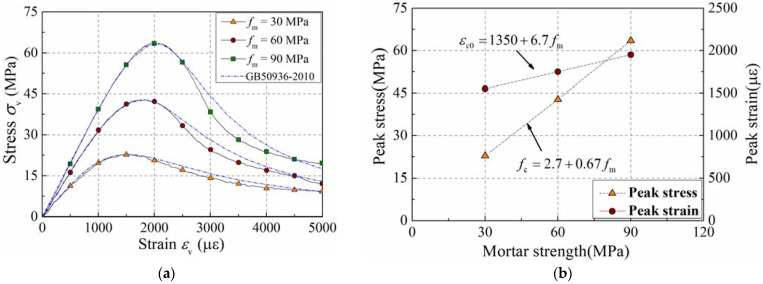
Influence of the mortar strength on performance of unconfined concrete. (**a**) Stress–strain curve; (**b**) strength relationship.

To illustrate the accuracy of the mesoscale model, the stress–strain curves of concrete columns were compared between the simulation results and specification results, as presented in Figure 3a. The comparison indicates that the simulation results coincide well with the specification results, and the mesoscopic model is an efficient tool for investigating the properties of plain concrete.

Figure 3b presents the strength relationship between concrete and mortar by analyzing the peak values of these curves. This figure indicates that both peak strain and peak stress of concrete are linearly related to the strength of the mortar phase, and the strength relationship is presented in Equation (5).

(5) $$f_{c}=2.7+0.67f_{m}$$

In this section, the numerical model of concrete with different strengths was established. The proposed model was efficient in describing the compressive stress–strain curve of unconfined concrete. Based on this model, it is valuable to study the dilation property of concrete in active confinement by applying the confining pressure.

## 3. Validation of the Mesoscale Model

### 3.1. Stress–Strain Curve

**Figure 4:**
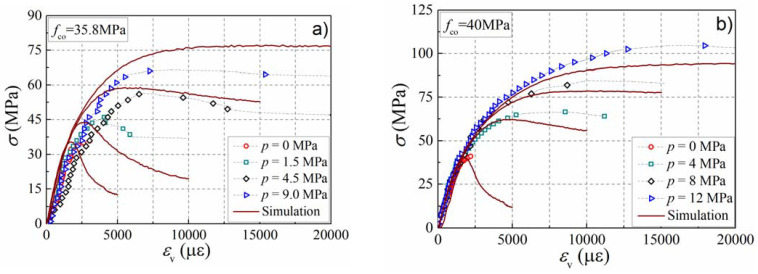

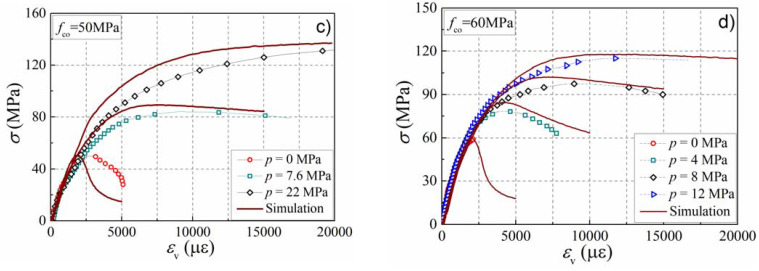
Comparison of the stress–strain curves between simulation and test results. (**a**) *f*_co_ = 35.8 MPa; (**b**) *f*_co_ = 40 MPa; (**c**) *f*_co_ = 50 MPa; (**d**) *f*_co_ = 60 MPa.

**Figure 5:**
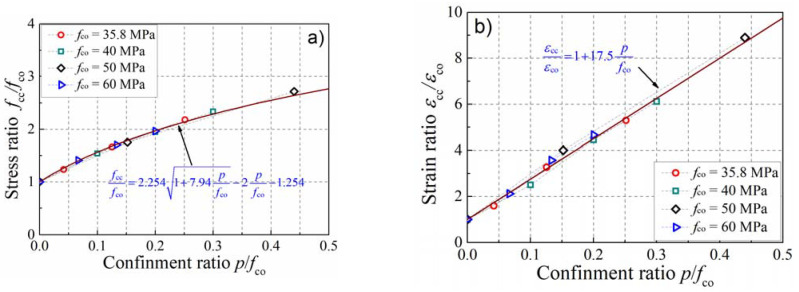
The enhancement of stress and strain in the simulation. (**a**) Stress enhancement; (**b**) strain enhancement.

In order to verify the prediction accuracy of the mesoscale model on the performance of actively confined concrete, concrete specimens with four strengths, such as 35.8, 40, 50, and 60 MPa, were collected from the literature [5,9,11] to create a comparison with the simulation results, as presented in Figure 4. The dotted lines represent the experimental stress–strain curves of concrete under different confinement levels, while the solid lines represent the simulation results of the mesoscale model. One can notice that the peak strain, peak stress, and the ductility of concrete are enhanced with the increase in the confinement level. The development of axial strain in the test is slightly faster than that in the simulation, and this may be connected with the sensitivity and discreteness of the strain measurement. Fortunately, the maximum stress on the stress–strain curve in the simulation was consistent with the test result. Moreover, the numerical model can also reflect the development trend of the stress–strain curve in the test. Figure 5 presents the influence of the confinement ratio on the enhancement of peak stress and peak strain. In the figure, the scatter points represent the peak stress or peak strain of the numerical simulation, while the solid lines represent the calculation results of theoretical formulas presented in the literature. With the increase in the confinement ratio, the growth rate of the stress ratio decreases and presents a nonlinear development trend. However, the strain ratio increases linearly with the increase in the confinement ratio. It can be observed that the simulation results agree well with the theoretical formulas describing the stress and strain enhancements, presented by Equations (6) and (7), which were introduced by Mander et al. [38] and Jiang et al. [4], respectively. The above comparison illustrates the validity of the numerical model in forecasting the mechanical behavior of concrete in active confinement.

(6) $$\frac{f_{\mathrm{cc}}}{f_{\mathrm{co}}}=2.254\sqrt{1+7.94\frac{p}{f_{\mathrm{co}}}}-2\frac{p}{f_{\mathrm{co}}}-1.254$$

(7) $$\frac{\varepsilon_{\mathrm{cc}}}{\varepsilon_{\mathrm{co}}}=1+17.5\frac{p}{f_{\mathrm{co}}}$$

### 3.2. Lateral–Axial Strain Curve

**Figure 6:**
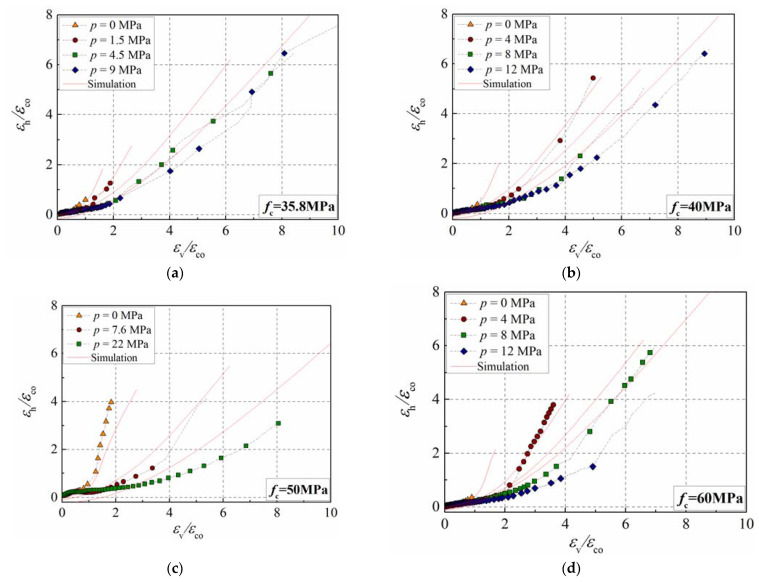
Comparison of the lateral–axial strain curves between simulation and test results. (**a**) *f*_co_ = 35.8 MPa; (**b**) *f*_co_ = 40 MPa; (**c**) *f*_co_ = 50 MPa; (**d**) *f*_co_ = 60 MPa.

The lateral–axial strain relationship is an important curve reflecting the dilation properties of concrete. Nevertheless, the strain measurement presents considerable discreteness due to the influence of the tester, equipment, and strain gauge displacement. The numerical model previously established above can overcome the shortcomings of tests and provide an effective means for studying concrete’s lateral–axial strain curve. Figure 6 compares the lateral–axial strain curves in the test and simulation. In the simulation, a two-step loading method was used. The confining pressure in the radial direction was first applied, and then the displacement in the longitudinal direction was applied. Therefore, the lateral strain was less than zero when the axial strain was zero. The figure presents that the lateral–axial strain curve tends to be flat with the increase in the confining pressure. Although some differences exist between the simulation and test results, the numerical model can reflect the development trend of the lateral–axial strain curve well. Thus, the mesoscale simulation can be regarded as an effective tool for studying the dilation properties of concrete in active confinement accounting for the sensitivity of the strain measurement.

The analysis described above shows that the proposed mesoscale model can make a good estimation on the stress–strain and lateral–axial strain curves of concrete in active confinement, illustrating that it is an efficient tool for investigating the mechanical and deformational behavior of concrete.

## 4. Parameter Analysis

To study the dilation properties of concrete in active confinement, the simulations of a concrete cylinder with different strengths and confinement ratios were conducted based on the mesoscale model. The strengths of the concrete cylinder were *f*_c_ = 30 MPa, 40 MPa, 50 MPa, and 60 MPa. Moreover, the confinement ratios were *p*/*f*_c_ = 0, 0.1 0.2, 0.3, and 0.4.

### 4.1. Failure Mode and Strain Distribution

**Figure 7:**
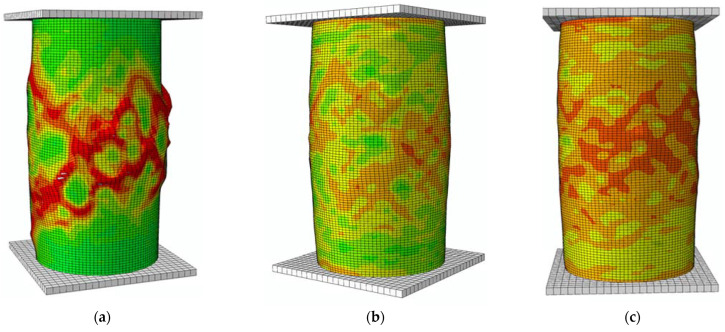
Influence of confinement ratio on the failure mode of a concrete cylinder. (**a**) *p*/*f*_co_ = 0; (**b**) *p*/*f*_co_ = 0.2; (**c**) *p*/*f*_co_ = 0.4.

The failure modes of the concrete cylinder having different confinement ratios are presented in Figure 7, and the concrete’s strength was 40 MPa. It can be observed from Figure 6 that shear failure occurs in the concrete cylinder with no confining pressure. With the increase in the confinement ratio, the specimen’s failure mode changes from shear failure to expansion failure, illustrating that the ductility of concrete increases with the development of confinement. The difference in failure modes indicates that the dilation of plain concrete is localized, while the dilation of concrete with a high confinement ratio is more homogeneous.

**Figure 8:**
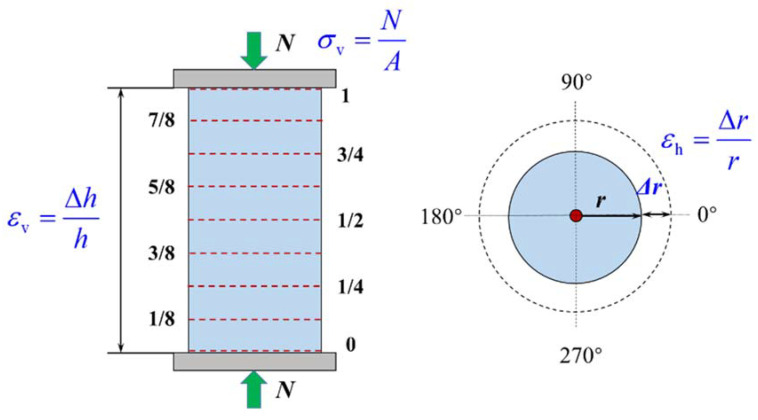
Schematic diagram of section location and strain calculation method.

The failure of the concrete cylinder with no or low confinement ratios was mainly concentrated in the middle of the specimen, where the dilation was the most significant; thus, the measurement of the lateral strain in the test was focused here. To investigate the strain distribution along the longitudinal direction, nine sections were selected along the specimen’s height in the simulation. The total height of the concrete cylinder was 200 mm, and the spacing between sections was 25 mm. Figure 8 presents the schematic diagram of the section location and strain calculation method.

**Figure 9:**
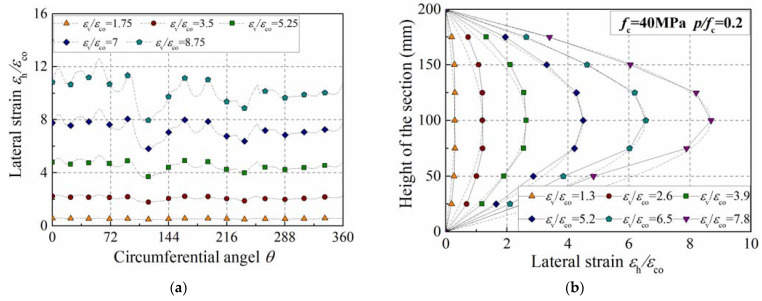
Distribution of lateral strain on concrete cylinder. (**a**) Influence of section angle; (**b**) influence of section height.

Figure 9a presents the distribution of the lateral strain in the middle section (1/2 height). It can be observed that the lateral strain is uniformly distributed in a circumferential direction as the axial strain ratio, $\varepsilon_{v}/\varepsilon_{\mathrm{co}}$, is set as 1.75 or 3.5. Nevertheless, the distribution of the lateral strain begins to become non-uniform as the axial strain ratio, $\varepsilon_{v}/\varepsilon_{\mathrm{co}}$, exceeds 5.25. The peak strain of the confined concrete is about 4.5 times that of plain concrete when the confinement ratio is set as 0.2 according to Equation (5), namely, $\varepsilon_{\mathrm{cc}}/\varepsilon_{\mathrm{co}}=4.5$. Thus, it can be concluded that the transverse dilation of concrete is not uniformly distributed as the axial strain exceeds the peak strain of confined concrete. A similar phenomenon was also observed in plain concrete in our previous work [39]. Figure 9b describes the development of lateral strain along the longitudinal direction; the concrete’s strength is 40 MPa and the confinement ratio is set as 0.2. It can be observed that the lateral strain is not uniformly distributed along the specimen’s height, which is almost in a sinusoidal form. 

**Figure 10:**
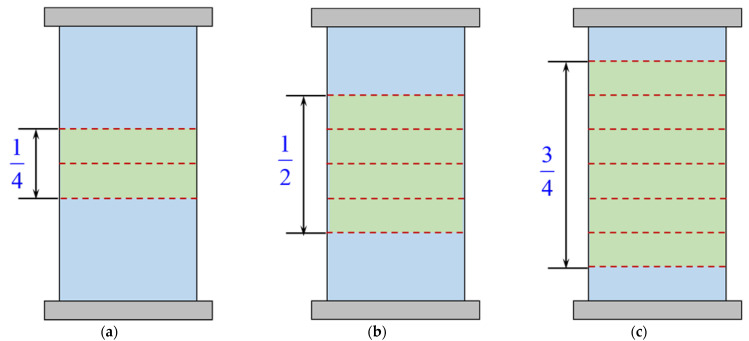
Average zone of the specimen with different confinement ratios. (**a**) *p*/*f*_co_ = 0 or 0.1; (**b**) *p*/*f*_co_ = 0.2; (**c**) *p*/*f*_co_ = 0.3 or 0.4.

Generally, the distribution of the lateral strain along the circumference and the height of the specimen is not uniform when compressive failure occurs in concrete. Therefore, great discreteness exists in the strain measurement in the tests. In order to reduce the influence of discreteness, the average strain was used in the analysis of the simulation results. Specifically, the lateral strain at each section is the average of 100 points along the circumferential direction, and the average strain at several sections can be regarded as the specimen’s lateral strain. Considering that the failure of the concrete cylinder with no or low confinement ratio was prone to local damage, while the failure of the specimen with a high confinement ratio was relatively uniform, the difference was considered in the current study when obtaining the average value. By trial and error and comparing with the test data, the authors observed that if 3, 5, and 7 sections were selected for the specimens with low (0 or 0.1), middle (0.2), and high (0.3 or 0.4) confinement ratios, respectively, presented in Figure 10, the simulation would produce a satisfactory estimation. The zone proportions were 1/4, 1/2, and 3/4 of the specimen height, and the average values were approximately 0.97, 0.90, and 0.78 of the maximum value in the middle section.

### 4.2. Secant Strain Ratio of The Specimen

**Figure 11:**
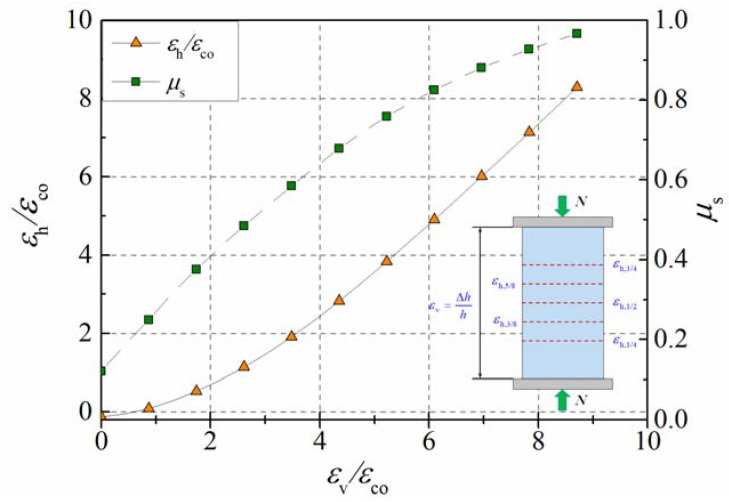
Transverse dilation of concrete cylinder with a confinement ratio of 0.2.

Figure 11 presents the development of the lateral strain of the specimen with a confinement ratio of 0.2, and the concrete’s strength is 40 MPa. Deformations in the lateral direction are usually described by the secant strain ratio, $\mu_{s}$ ($\mu_{s}=\varepsilon_{h}/\varepsilon_{v}$), which is also plotted in this figure. In the elastic stage, the secant strain ratio of unconfined concrete equaled the Poisson’s ratio, ranging from 0.15 to 0.20. The initial secant strain ratio of the specimen mentioned above was about 0.12, which was slightly less than the Poisson’s ratio of plain concrete, because the secant strain ratio is influenced by confining pressure. It should be noticed that the growth rate gradually decreased with the development of the axial strain. The strength grade and confinement ratio were important parameters affecting the properties of the concrete, thus, the influences of these two parameters on the dilation properties of the concrete in active confinement were analyzed in the following sections.

### 4.3. Influence of the Compressive Strength

**Figure 12:**
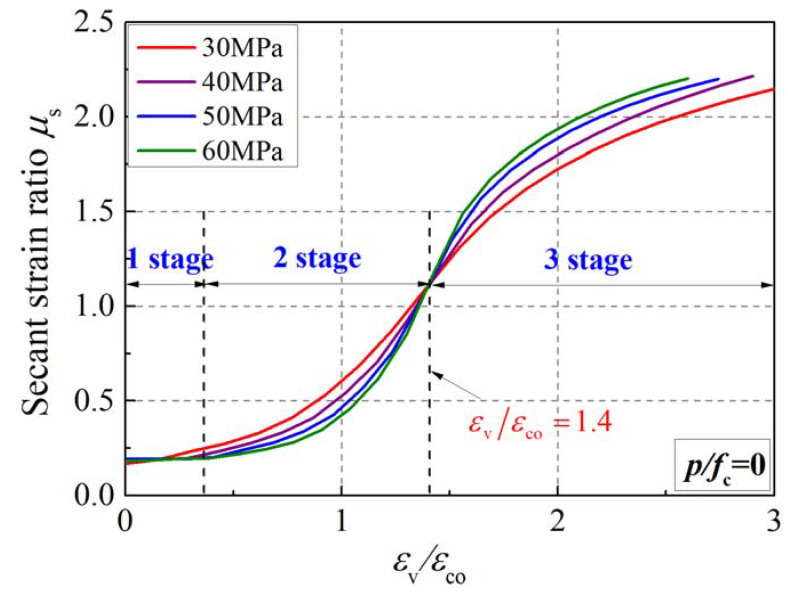
Influence of strength grade on secant strain of plain concrete.

Figure 12 presents the influence of strength grade on the secant strain ratio of plain concrete. It can be observed that the development of the secant strain ratio is generally divided into three stages. The secant strain ratio remains constant in the first stage and equals the Poisson’s ratio of the concrete, as the micro-cracks slowly develop in this stage. It should be noticed that the platform segment was affected by the strength of the concrete; the higher the strength, the wider the platform segment. This was because the high-strength concrete had a greater range of elastic segments. In the second stage, the value and the growth rate continue to increase as the axial strain increases due to the accumulation of internal cracks. As the cracks develop slowly in the high-strength concrete specimen, the secant strain ratio is relatively smaller. In the third stage, the growth rate gradually decreases, although the secant strain ratio continues to increase. Due to the brittleness of the high-strength concrete, its secant strain ratio becomes larger than that of the low-strength concrete in this stage. The secant strain ratio is about 0.5 as the axial strain approaches the peak strain, which was demonstrated by the studies in the literature [3,7,40].

**Figure 13:**
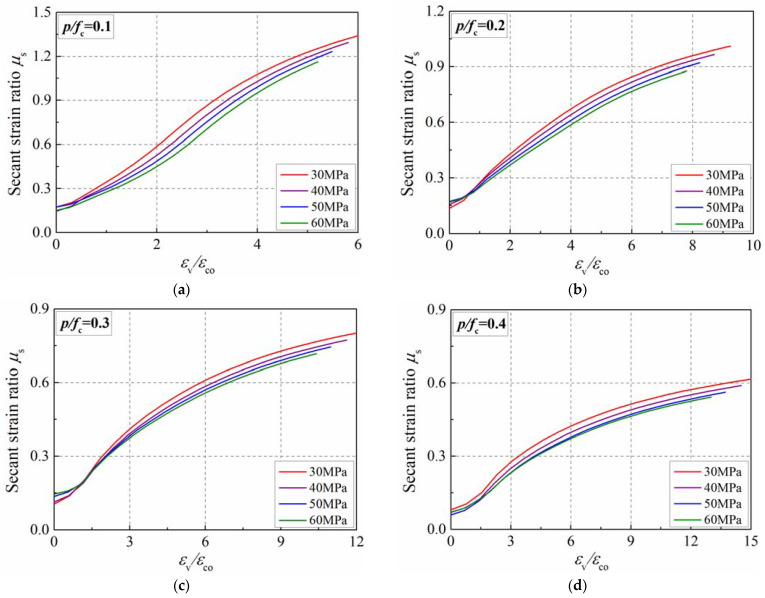
Influence of strength grade on secant strain ratio of concrete in active confinement. (**a**) *p*/*f*_c_ = 0.1; (**b**) *p*/*f*_c_ = 0.2; (**c**) *p*/*f*_c_ = 0.3; (**d**) *p*/*f*_c_ = 0.4.

Figure 13 reflects the influence of strength grade on the secant strain ratio of concrete in active confinement. One can observe that its value continues to increase as the axial strain increases. Similar to plain concrete, the growth rate of the secant strain ratio of actively confined concrete increases during the early stage, and slows down later on. The influence of strength grade on the secant strain ratio of concrete in active confinement is slightly different from that of plain concrete. Specifically, the influence of strength on the secant strain ratio of confined concrete is monotonic. The greater the strength, the smaller the secant strain ratio. This is because the greater the concrete’s strength, the higher the confining pressure as the confinement ratio is constant, inhibiting the brittle failure of the high-strength concrete.

Overall, the strength grade influenced the development of concrete’s secant strain ratio, especially for concrete specimens with no or low confinement ratios.

### 4.4. Influence of the Confinement Ratio

**Figure 14:**
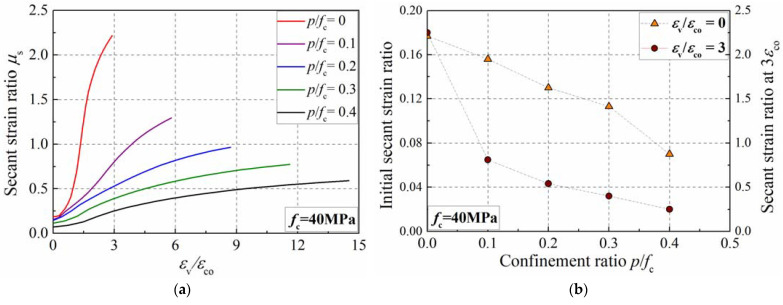
Influence of confinement ratio on secant strain ratio of concrete cylinder. (**a**) Development of secant strain ratio; (**b**) secant strain ratio at zero strain and 3ε_co_.

The influence of the confinement ratio on the dilation of the specimen with different strengths is similar. Figure 14a presents the simulation results of the concrete specimen with a strength of 40 MPa. With the increase in the confinement ratio, the transverse dilation is inhibited, and then the secant strain ratio slowly develops. Figure 14b presents the influence of the confinement ratio on the secant strain ratio at zero strain and 3*ε*_co_. When the axial strain is three times the peak strain of plain concrete, the secant strain ratio is 2.25, 0.81, 0.54, 0.40, and 0.25 as the confinement ratio is set as 0, 0.1, 0.2, 0.3, and 0.4, respectively. Moreover, the initial secant strain ratio is 0.177, 0.156, 0.130, 0.113, and 0.070 when the confinement ratio increases from 0 to 0.4, illustrating that the confining pressure can inhibit the development of the secant strain ratio. The analysis indicates that the confinement ratio is an important parameter affecting the transverse dilation of concrete, which should be considered in the formula.

### 4.5. Dilation Model for Actively Confined Concrete

**Figure 15:**
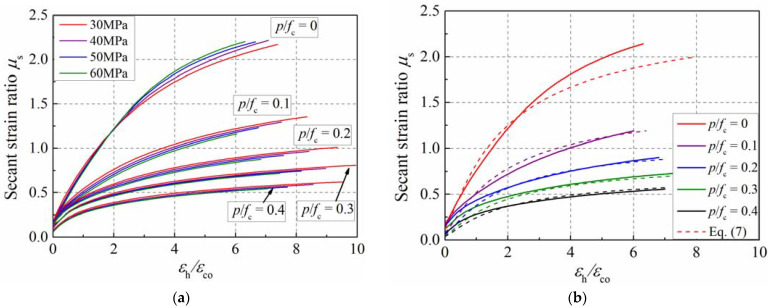
Influence of strength grade and confinement ratio on secant strain ratio. (**a**) Strength grade; (**b**) confinement ratio.

The simulation results show that the confinement ratio has a more significant influence on transverse dilation than that of concrete’s strength, as presented in Figure 15a. Considering the weak effect of concrete’s strength, the average curves were adopted to establish the dilation formula of actively confined concrete, which is presented in Figure 15b. The dotted lines with colors of red, purple, blue, green and black in Figure 15b represent the equation results for the specimens with confinement ratios of 0, 0.1, 0.2, 0.3 and 0.4, respectively. The key to predicting the secant strain ratio lies in the establishment of the lateral–axial strain relationship of concrete. Moreover, it is crucial to express the axial strain as an explicit function of the lateral strain, because it is more convenient to calculate the confining pressure and axial strain. Otherwise, it is not feasible.

**Figure 16:**
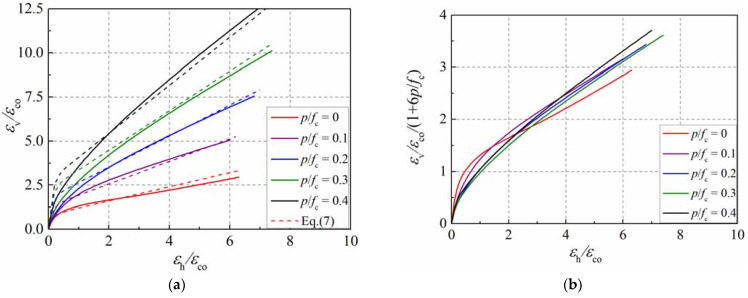
Lateral–axial strain curves of concrete with varying confinement ratios. (**a**) Lateral–axial strain curves; (**b**) equivalent lateral–axial strain curves.

Figure 16a presents the lateral–axial strain curves of concrete cylinders with varying confinement ratios. The dotted lines with colors of red, purple, blue, green and black in Figure 16a represent the equation results for the specimens with confinement ratios of 0, 0.1, 0.2, 0.3 and 0.4, respectively. According to the study in the literature [13], the following expression was adopted to describe the lateral–axial strain relationship of concrete in active confinement, as presented by Equation (8).

(8) $$\frac{\varepsilon_{v}}{\varepsilon_{\mathrm{co}}}=\left(\alpha +\beta \frac{p}{f_{c}}\right)A\left\{{\left[1+B\left(\frac{\varepsilon_{h}}{\varepsilon_{\mathrm{co}}}\right)\right]}^{C}-\exp\left[D\left(\frac{\varepsilon_{h}}{\varepsilon_{\mathrm{co}}}\right)\right]\right\}$$

As the formula can apply to unconfined concrete, the value of parameter *α* equals 1.0. The confinement ratio *p*/*f*_c_ can be set as zero for unconfined concrete. Previous studies [3,7,39] have shown that the secant strain ratio is approximately 0.5 as the axial strain approaches the peak strain of unconfined concrete, which needs to be considered in the formula. The coefficients A, B, C, and D can be determined according to the simulation results of unconfined concrete. By analyzing the lateral–axial strain curve in the simulation, the coefficients A, B, C, and D equal to 0.8, 0.5, 1.0, and −9, respectively. Coefficient *β* reflects the influence of the confinement ratio, and a satisfactory prediction can be achieved when it is set as 6.0. Figure 16b presents the equivalent lateral–axial strain relationship of the concrete cylinder. The curves present a slight difference after considering the equivalent reduction in the confinement ratio. Therefore, the final expression of the lateral–axial strain relationship of the concrete in active confinement is presented by Equation (9).

(9) $$\frac{\varepsilon_{v}}{\varepsilon_{\mathrm{co}}}=0.8\left(1+6\frac{p}{f_{c}}\right)\left\{1+0.5\left(\frac{\varepsilon_{h}}{\varepsilon_{\mathrm{co}}}\right)-\exp\left[-9\left(\frac{\varepsilon_{h}}{\varepsilon_{\mathrm{co}}}\right)\right]\right\}$$

**Figure 17:**
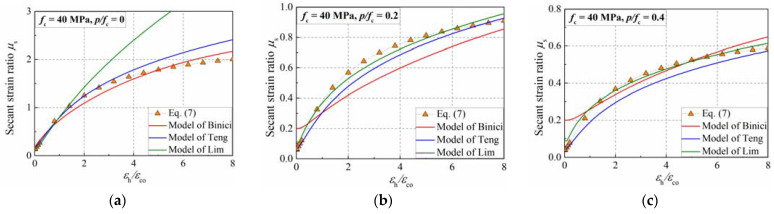
Secant strain ratio prediction of the proposed and existing formulas. (**a**) *p*/*f*_c_ = 0; (**b**) *p*/*f*_c_ = 0.2; (**c**) *p*/*f*_c_ = 0.4.

To illustrate the performance of the proposed lateral strain equation, it was compared with the simulation results presented in Figure 15b and Figure 16a. Moreover, the lateral–axial strain curves proposed by Binici et al. [3], Teng et al. [13], and Lim et al. [14] were utilized to have a comparison, as shown in Figure 17. It can be observed that Lim’s model can produce a good estimation of the dilation of confined concrete, but it overestimates the dilation of plain concrete. Although Binici’s model can reflect the dilation behavior of the concrete cylinder, the influence of the confinement ratio on the initial secant strain ratio was not considered. Teng’s model can produce a good estimation of the development of the secant strain ration in the simulation.

In general, the proposed lateral strain formula can reflect the simulation results of the mesoscale model, and it is consistent with the prediction of the existing formulas. Therefore, it is an effective formula for predicting the dilation properties of actively confined concrete.

## 5. Conclusions

A mesoscale concrete model was established to investigate the dilation properties of actively confined concrete in the current study. The following conclusions were formulated through the analysis:

1. The simulation results of the mesoscale model were in good agreement with the stress–strain curves of the plain concrete from codes, based on which the strength relationship between the concrete and mortar was established.
2. The mesoscale model made a good estimation of the stress–strain and lateral–axial strain curves of the concrete in active confinement, illustrating that it was an efficient tool for investigating the concrete’s mechanical and deformational behavior.
3. The distribution of the lateral strain along the circumference and the height of the specimen was not uniform when compressive failure occurred in the concrete.
4. The confinement ratio had a more significant influence on the transverse dilation than that of the concrete’s strength. With the increase in the confinement ratio, the transverse dilation was inhibited, and then the concrete’s secant strain ratio slowly developed.
5. An expression of the lateral–axial strain curve of the concrete in active confinement was proposed, which can reflect the simulation results of the mesoscale model, and it was consistent with the prediction of the existing formulas.

Mesoscopic analysis can reflect the heterogeneity of concrete and the interaction among different phases, which is an efficient method to study the mechanical and deformation behavior of concrete. At present, the content of our research is limited, and we anticipate conducting further research using this approach in the future, such as the influence of nano silica or fiber on the performance of concrete, and the interaction mechanisms of the steel tube and concrete core.

## Figures and Tables

**Table 1:** Parameters used in the mesoscale model.

	Aggregate	Mortar Matrix	ITZ
Mass density (kg/m^3^)	2400	2400	2400
Compressive strength	-	*f*_m_	0.8 *f*_m_
Tensile strength	-	$1.4{\left(f_{m}/10\right)}^{2/3}$	$1.12{\left(f_{m}/10\right)}^{2/3}$
Elastic modulus (GPa)	60	$4000\sqrt{f_{m}}$	$3200\sqrt{f_{m}}$
Fracture energy (N/mm)	-	$G_{F0}{\left(f_{m}/10\right)}^{0.7}$	$0.8G_{F0}{\left(f_{m}/10\right)}^{0.7}$
Poisson’s ratio	0.20	0.20	0.20

## Data Availability

Not applicable.
